# Electron Irradiation Effects on Strength and Ductility of Polymer Foils Studied by Femtosecond Laser-Processed Micro-Tensile Specimens

**DOI:** 10.3390/ma12091468

**Published:** 2019-05-07

**Authors:** Manuel J. Pfeifenberger, Gabor Milassin, Anton Hohenwarter, Barbara Putz, Christopher O. A. Semprimoschnig, Reinhard Pippan

**Affiliations:** 1 Erich Schmid Institute of Materials Science, Austrian Academy of Sciences, 8700 Leoben, Austria; barbara.putz@empa.ch (B.P.); reinhard.pippan@oeaw.ac.at (R.P.); 2 ESA-ESTEC (TEC-QEE), Keplerlaan 1, 2201 AZ Noordwijk, The Netherlands; gabor.Milassin@esa.int (G.M.); Christopher.Semprimoschnig@esa.int (C.O.A.S.); 3 Department of Materials Science, Chair of Materials Physics, University of Leoben, 8700 Leoben, Austria; anton.hohenwarter@unileoben.ac.at

**Keywords:** micro tensile testing, ultrashort pulse laser ablation, electron beam irradiation, sample preparation, polymer foil, local mechanical properties

## Abstract

The influence of irradiation on mechanical properties of polymer foils used in spacecraft applications has widely been studied via macroscopic tensile samples. An increase in the local resolution of this investigation can be achieved by reducing the sample’s dimensions. A femtosecond laser enables a fast fabrication of micro-samples with dimensions from tens of μm to the mm range, with ideally no influence on the material. Tensile experiments using such micro-tensile samples were conducted on FEP, Upilex-S and PET foils. The influence of the laser processing on the polymer foils was evaluated. Additionally an investigation of degradation due to electron irradiation was performed. Furthermore an outlook to extend this technique to depth-resolved measurements by preparing samples from locally thinned foils is presented. The study demonstrates the feasibility of femtosecond laser processing for rapid fabrication of micro-samples, enabling insights into the effect of electron irradiation on local mechanical properties of polymers.

## 1. Introduction

Polymer foils used in spacecrafts, e.g., as thermal control foils, are usually exposed to multiple types of radiation and severe thermal cyclic loading [1,2,3]. The influence of irradiation on the mechanical properties of polymer foil materials has already been studied widely, mainly via testing macroscopic tensile specimens [1,4,5,6,7,8,9,10]. Existing experiments on the macro scale cannot, however, resolve local variations in mechanical properties, e.g., caused by degradation due to inhomogeneous irradiation conditions. The mapping of such variations requires a significant reduction of the sample dimensions, compared to the dimensions specified in ASTM D882 [11]. Very small specimens can additionally contribute to an efficient investigation of materials which are limited in their availability or exclusive in their kind, e.g., polymer foils retrieved from space missions.

A femtosecond (fs) laser enables a fast and reproducible way for the fabrication of such micro-samples. This technique offers optimal properties for material processing on a scale from tens of micrometers up to a few millimeters. Most importantly, using a fs-laser for processing impedes melting of the specimen material during the ablation due to ideally no thermal diffusion into the material. This allows precise and reproducible fabrication of structures on the micro-scale [12]. An increase in processing precision, compared to lasers with a longer pulse duration, has been found for various classes of materials including polymers, semiconductors and ceramics [13,14]. The negligible material modification during fabrication of the sample is a significant advantage compared to other techniques for small scale processing like micro electric discharge machining [15] or laser processing using lasers with pulse durations above the ultra-short pulse regime (i.e., >10 ps) [16]. However, the high material removal rate of the fs-laser competes with the rate of the aforementioned techniques and outperforms other methods such as the focused ion beam (FIB) or Plasma FIB by orders of magnitude [17]. The presented features make the fs-laser particularly interesting for the micro-processing of polymers.

The processing of micro tensile samples by means of a fs-laser has rarely been studied. Works by Slaughter et al. [18], Severin et al. [19] and Magagnosc et al. [20] already showed the feasibility of this method for micro tensile sample fabrication of different materials. A proper investigation of the laser processing influence on the mechanical properties of polymers is missing, though. Hence, in this study, three types of polymers with strongly different mechanical properties have been investigated and the results are discussed in comparison with macroscopic results from literature.

The polymer foils were exposed to electron irradiation with typical dose levels seen by spacecraft in the low earth orbit to asses the capability of the micro-tensile experiments to study the influence on the material. Other influencing factors of the space environment like, thermal cycling, high vacuum, electromagnetic irradiation or further types of charged particle irradiation [9,21] have not been investigated in this work. The influence of mixed irradiation is discussed elsewhere [1,2,3,5,6,8].

Micro tensile specimens allow to evaluate the mechanical properties on a scale of a few hundred micrometer, hence, this study provides a technique, which can yield insights into the local irradiation damage of polymer foils. Additionally, the determination of damage gradients across the thickness of polymer foils can be of interest. It is particularly interesting to investigate the depth of embrittlement zones due to (space) irradiation. The fs-laser processing combined with the capability of precise positioning allows to locally slice the foils into thinner sections. This depicts a promising technique to enable the acquisition of depth-resolved mechanical data.

This study is structured as follows: The first part deals with the experimental details of the work. In Section 3.1 results on the influence of the laser processing on the polymer foils are given and discussed. The results of the micro-sample tensile experiments are shown in Section 3.2. Section 3.3 contains the investigation of the influence of electron irradiation doses present in space environment conditions, on the mechanical properties of the foils. Details on the effect of different ambient atmospheric conditions during the laser processing on the materials are discussed in Section 3.4. Finally, an outlook on the fabrication of tensile samples with varying thickness, via local thinning of the foils, is presented.

## 2. Experimental

In this study a recently developed system, which combines a fs-laser with a FIB and a scanning electron microscope (SEM) was used (based on the Auriga Laser platform—Zeiss, Oberkochen, Germany). Details of the system can be found in a previous study [22]. The integrated fs-laser is a Origami 10 XP (Onefive GmbH, Regensdorf, Switzerland). The fs-laser has a pulse duration of about 500 fs, two available wavelengths of 1030 and 515 nm and a maximum pulse repetition rate of 1 MHz. For the sample processing the wavelength of λ=515 nm, which allows a maximum average output power of about 2 W, is used. Two lenses placed prior to the scan unit act as beam expander. Their distance determines the focal height in the processing chamber. The beam is guided by a galvanometer scan unit (intelliscan III 10, SCANLAB AG, Puchheim, Germany). The objective lens allows an optimal focal diameter at the sample surface of approximately 25μm. SEM imaging is used to evaluate the quality of the laser processed samples.

### 2.1. Materials

Three different polymer foil materials were investigated in this work:

- Polyimide Upilex-S VDA (25.4 μm-thickness) from UBE
- PET (Polyethylene terephthalate) (127 μm) from Sheldahl
- FEP (Fluorinated ethylene propylene) VDA (127 μm) from Sheldahl

The Upilex-S and FEP foil are coated with a vapor deposited aluminium (VDA) thinfilm (with 100 nm-thickness) on one side. The PET foil is not coated.

The three types of polymer foil materials exhibit distinctively different tensile properties, ranging from high ultimate strength to high ultimate strain, therefore, this materials are ideally suited to assess experiments with fs-laser fabricated micro-tensile specimens. Furthermore, Upilex-S VDA and FEP VDA foils are commonly used in multilayer insulation of spacecraft [21,23], hence, an evaluation of their resistivity against electron irradiation is of essential interest.

#### Electron Irradiation Procedure

The electron irradiation doses of the outer layer of thermal blankets of the Hubble Space Telescope in the low Earth orbit roughly range from 100 kGy to about 1 MGy for the shortest servicing missions up to the defined end-of-life duration respectively [2,24]. The electron irradiation doses investigated in this study cover this dose range.

The irradiation was performed in a van de Graaff-type electron accelerator producing an electron beam with 1MeV and a dose rate of 146Gy/s. The 2 centimeter thick aluminium exposure table was water-cooled and a nitrogen flushed frame was placed above the samples to keep them close to room temperature and to inhibit oxidation. For the dosimetry measurements radiochromic Nylon thin foil dosimeters were used. Coated foils were irradiated from the non-coated side. Material samples were grouped in stacks of different heights on the exposure table, 12 foils for Upilex-S VDA, 4 foils for FEP VDA and 4 foils for PET. Due to the high electron energies, the small total stack thickness and the low density of the polymers, the dose rate was assumed to be constant across the thickness during the whole exposure process. In order to reach different irradiation dose levels, the exposure was interrupted regularly to remove foils from the stack. Hence, for example, to achieve a total irradiation dose of 250 kGy the foils were removed after 28.5 min from the exposure setup. For each material this procedure yielded samples with certain total electron irradiation doses (listed in Table 1). The FEP VDA foils became too brittle to handle for irradiation doses above 250 kGy, hence, only two irradiation dose levels were investigated for this material.

### 2.2. Methods

#### 2.2.1. Laser Processing

The focal plane was determined by selecting the ablation spot with minimum diameter via varying the distance of the beam expander lenses. The samples were placed in the middle of the scan field to ensure a normal incidence of the laser beam onto the foil.

In Table 2 the laser processing parameters used for the fabrication of the tested specimens are listed. The laser processing parameters mainly responsible for the quality of the cut are the pulse repetition rate and the fluence. In Section 3.1 the influence of the laser pulse repetition rate on the sample quality is discussed in more detail. The fluence is the energy density at the focal spot and directly proportional to the maximum average power. For the investigated types of polymers (Polyimide, FEP and PET) multi-pulse fluence ablation thresholds of ultra-short pulsed laser were reported to be in the range of about 0.3–0.5 J/cm${}^{2}$ [14,25]. The fluence values given in Table 2, were selected close to the ablation threshold to ensure a high quality cutting surface, however, still being high enough to enable an adequate removal rate for a fast sample processing. The value for the Upilex-S VDA material is lower due to its smaller thickness.

**Figure 1:**
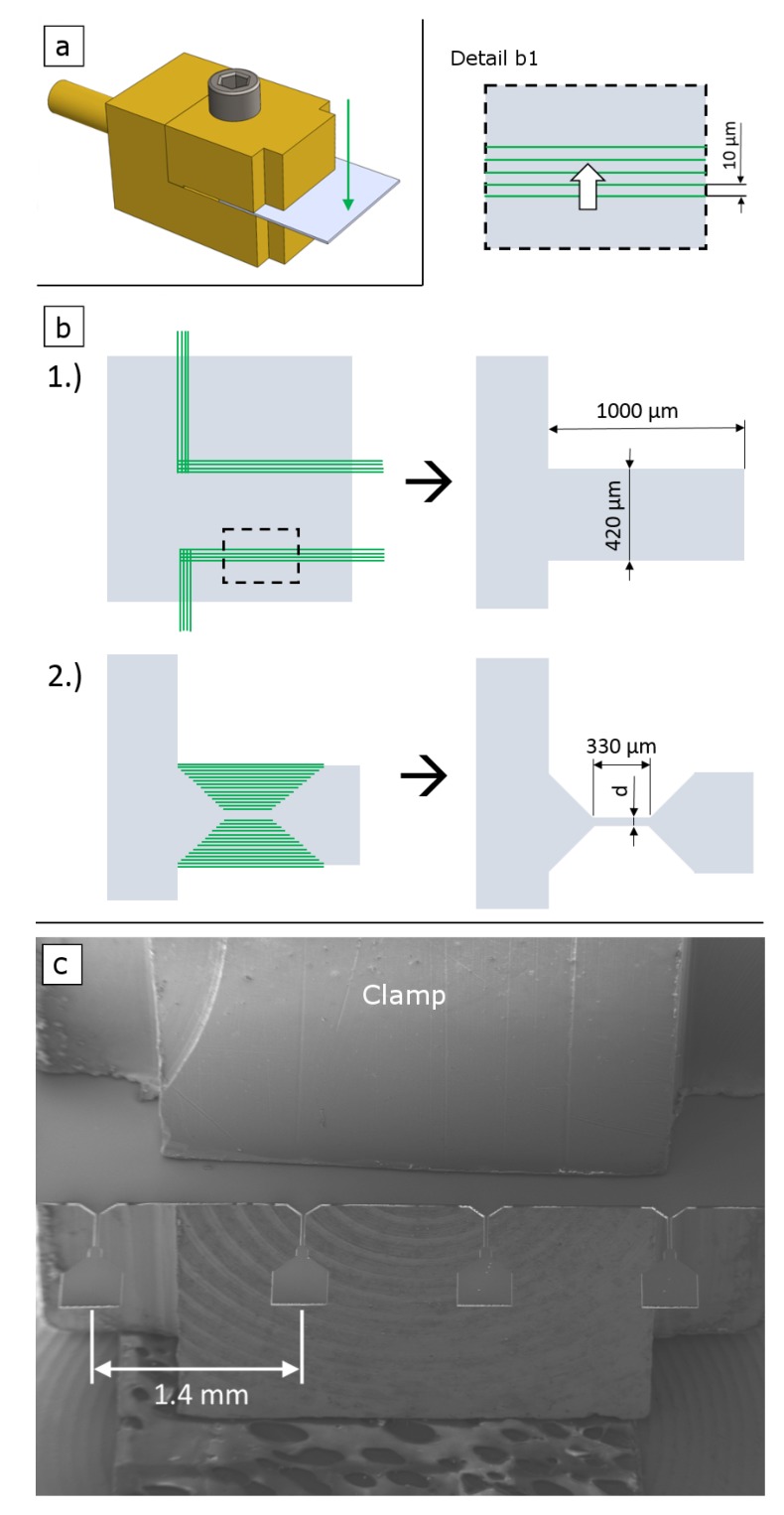
(**a**) 3D sketch of a foil fixed in a clamp with the VDA film on top. The arrow indicates the incidence direction of the laser beam. (**b**) Outline of the two step cutting process from the view of the laser incidence direction. First a rectangular shape with the intended width of the tensile head is cut. In the second step the dog bone shape is processed. Relevant dimensions of the specimen are given. The width *d* of the gauge section was about 100 μm for the FEP VDA and about 25 μm for the Upilex-S VDA foils. Detail (b1) displays the magnification of the dashed rectangle in step 1. Each laser scan line is shifted 10 μm in the direction of the broad arrow. (**c**) SEM image showing a set of four samples cut in a Upilex-S VDA foil, fixed in the clamp for the following tensile experiments.

The scan repetitions define how often each laser pattern, shown in Figure 1b, is successively scanned. This adds up to the total processing times listed in the table for the respective parameters of each sample material. The scanning strategy plays an important role, especially regarding the redeposit of ablated material. Further details regarding the scanning strategy can be found in [22]. If not stated otherwise, the fs-laser processing was conducted under a vacuum pressure of ca. $7\times 10^{-3}\;$mbar.

Prior to the laser processing a piece of the foil with a size of about 6×6 mm, cut using scissors, was fixed in a clamp, which could be afterwards mounted in the tensile testing device. The foils were fixed with the VDA side facing upwards. This is sketched in Figure 1a. The image also schematically indicates the laser beam incidence direction. Subsequently, the tensile geometry was cut in a two-step process as shown in Figure 1b. First a rectangular shape and second the dog bone shape was fabricated. The scan patterns consist of single lines successively shifted by a distance of 10μm (see detail in Figure 1b). The tensile samples are placed in a distance of 1.4 mm to each other to ensure enough space for the testing gripper. This enables to test a maximum of four samples on each piece of foil, fixed in a clamp (as shown in Figure 1c).

The final cross-section geometry of the samples shows a trapezoidal shape due to a certain taper angle resulting from the laser processing. The resulting mean values of the taper angles for the respective processing parameters of each sample material are listed in Table 2. This taper can not be compensated in the used fabrication setup, however, it shows good reproducibility when using the same processing parameters. The trapezoidal shape of the cross-section was taken into account for the stress evaluation of the tensile experiments.

#### 2.2.2. Setup of Tensile Experiments

The uni-axial tensile experiments were performed on a Kammrath & Weiss fibre tensile module [26]. This device enables to measure forces up to about 2N with a resolution of approximately 10μN. The displacement is registered with an accuracy of about 30nm. The experiments are recorded using a camera connected to a stereo microscope, taking images every five seconds. Using a moveable stage the sample can be properly aligned with the gripper. The gripper was cut out of a 100μm-thick tungsten foil, resembling the negative shape of the tensile sample head, with the same fs-laser system. All experiments were performed displacement controlled and the force was recorded. The testing speed was 1 μm/s, resulting in an initial strain rate of about 0.3%/s. Only the non-irradiated FEP VDA foils, due to their very high maximal strain values, were tested with 2 μm/s (strain rate 0.6%/s) to keep the test duration limited. This low testing speed values enable the capturing of enough pictures throughout the loading process to perform a continuous displacement correction of the data. Each sample was elongated until failure. The experiments were conducted at room temperature (22 ${}^{\circ }$C) under atmospheric conditions.

## 3. Results and Discussion

### 3.1. Influence of fs-Laser Processing

Femtosecond laser processing is a fast and versatile method for the fabrication of micro-samples, nevertheless, especially in the case of polymers the influence of heat and effects due to air ionization (see Section 3.4) need to be considered.

Specifically, in the case of polymers the repetition rate needs to be carefully chosen. The excessive energy of each laser pulse, which is not used to ablate material generates a small amount of heat [27]. If the pulse repetition rate is high, the accumulation of the heat can lead to a local melting of the processed materials [28]. Especially for materials with a low heat conductivity this is critical in terms of achieving a high quality of the cut.

**Figure 2:**
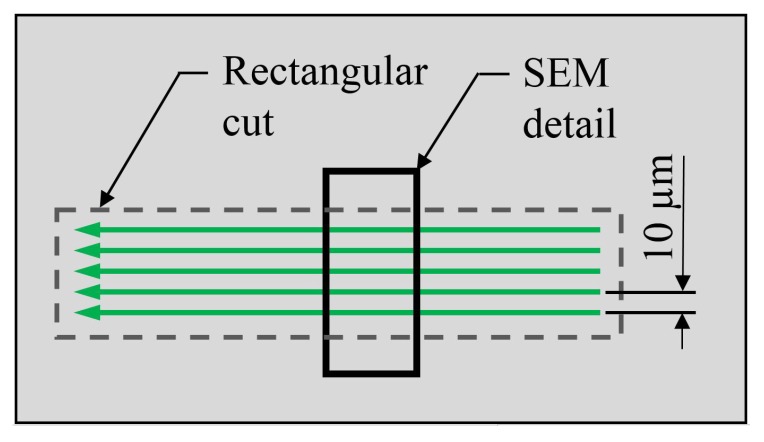
Scan pattern for the laser parameter tests. 5 parallel lines, each shifted for 10 μm, were cut into the foil and yielded a rectangular cut as indicated by the dashed line. The position of the SEM details shown in Figure 3, Figure 4 and Figure 5 is indicated.

**Figure 3:**
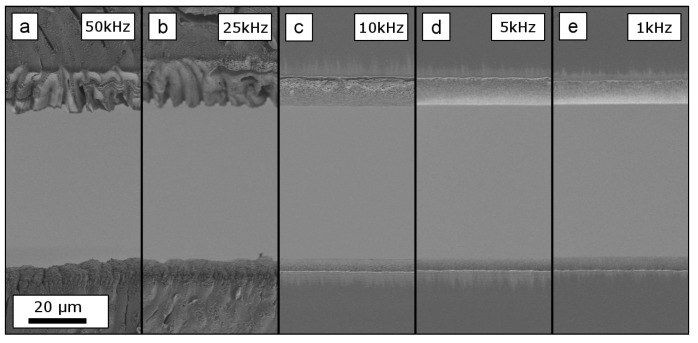
SEM details, as indicated in Figure 2, show the edges above and below the central rectangular cut in a 25.4μm-thick Upilex-S VDA foil. The cuts were processed with a constant total energy input, hence, decreasing pulse repetition rates whilst increasing the number of scan repetitions (corresponding to increasing machining times). (**a**,**b**) show massive damage, cracks and molten structures. In (**c**) local molten areas are observable on the upper edge. In (**d**,**e**) both edges exhibit clean cutting surfaces. The scale bar indicated in (**a**) applies to all figures.

**Figure 4:**
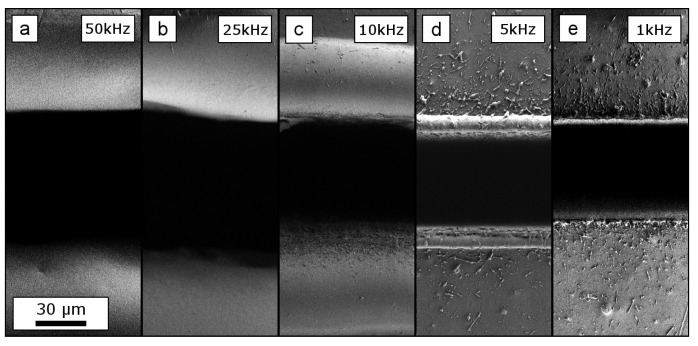
Upper and lower edge of rectangle cuts in a 127μm-thick PET foil. The cuts were processed with a constant total energy input, hence, decreasing pulse repetition rates ((**a**) 50 kHz; (**b**) 25 kHz; (**c**) 10 kHz; (**d**) 5 kHz; (**e**) 1 kHz) whilst increasing the number of scan repetitions (corresponding to increasing machining times). The foil thickness is cut through for all tested parameter combinations. Massive melting is found for repetition rates larger than 5 kHz. Note the larger scale bar, compared to Figure 3 and Figure 5. The scale bar indicated in (**a**) applies to all figures.

**Figure 5:**
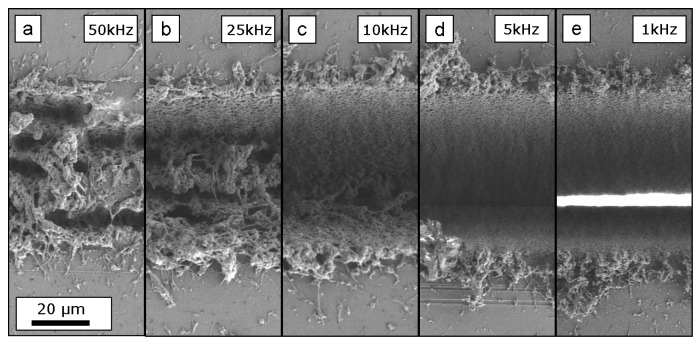
Upper and lower edge of rectangle cuts in a 127μm-thick FEP VDA foil. The cuts were processed with a constant total energy input, hence, decreasing pulse repetition rates ((**a**) 50 kHz; (**b**) 25 kHz; (**c**) 10 kHz; (**d**) 5 kHz; (**e**) 1 kHz) whilst increasing the number of scan repetitions (corresponding to increasing machining times). The foil thickness is cut through only for 50kHz and 50 scan repetitions as multiple scan repetitions enable more efficient material removal, compared to few scan repetitions and high pulse repetition rates. The scale bar indicated in (**a**) applies to all figures.

To evaluate the heat accumulation effect on the sample quality an experiment, varying the time of energy input, was performed on the three investigated materials. For this, five parallel lines with a length of 600 μm placed at a distance of 10μm were cut into the foil, resulting in a rectangular cut. This is sketched in Figure 2. The fluence was chosen to be 1.18 J/cm${}^{2}$, the scan speed 2mm/s and the laser pulse repetition rate was varied: 50,25,10,5,1kHz. To keep the total energy input constant the scan repetitions were adapted accordingly: 1,2,5,10,50 repetitions. Hence, the amount of laser pulses per cut are kept constant, but the processing duration varies from 1.5s to 75s. SEM details of the upper and lower edge of this rectangular cuts, as indicated in Figure 2, are shown in Figure 3 for Upilex-S VDA, in Figure 4 for PET and in Figure 5 for FEP VDA.

The 25μm-thick Upilex-S VDA foils were cut through the entire thickness for all parameter combinations. For pulse repetition rates of 50 and 25kHz, the specimens exhibited a massive damage up to a distance of 200μm around the processed area. For the pulse repetition rate of 10kHz (Figure 3c) still a small part of molten material was observable. For the cuts done with 5kHz and 1kHz clean cutting surfaces were obtained.

Pronounced melting was also found for the PET foils up to 150μm away from the cutting edge for the highest pulse repetition rates of 50 and 25kHz. For this material a molten burr also appeared for 5kHz. A pulse repetition rate of 1kHz again showed minimized damage. All pulse repetition rates allowed a cut through the entire 127μm-thick PET foils.

The damage of the FEP VDA foils showed less signs of damage due to melting, but an increase in debris deposition next to the processed structure compared to Upilex-S VDA and PET foils. Additionally, another critical issue occurred when compared to Upilex-S VDA and PET films. The use of high pulse repetition rates and respective small numbers of scan repetitions yields a small material removal rate. Therefore, a cut through the 127μm-thick FEP VDA foil, was only achieved for 50 scan repetitions using a pulse repetition rate of 1kHz (see Figure 5e). The good heat resistance of FEP experienced in the fs-laser cutting experiments agrees with the results of thermally cycled FEP, which did not exhibit significant changes in its tensile properties [7].

According to the results of these experiments on the variation of the pulse repetition rate, a rate of 1kHz was used for the fabrication of all tensile test specimens. The scan repetitions were set to values, which ensure a through thickness cut (see Table 2).

### 3.2. Tensile Experiments

**Figure 6:**
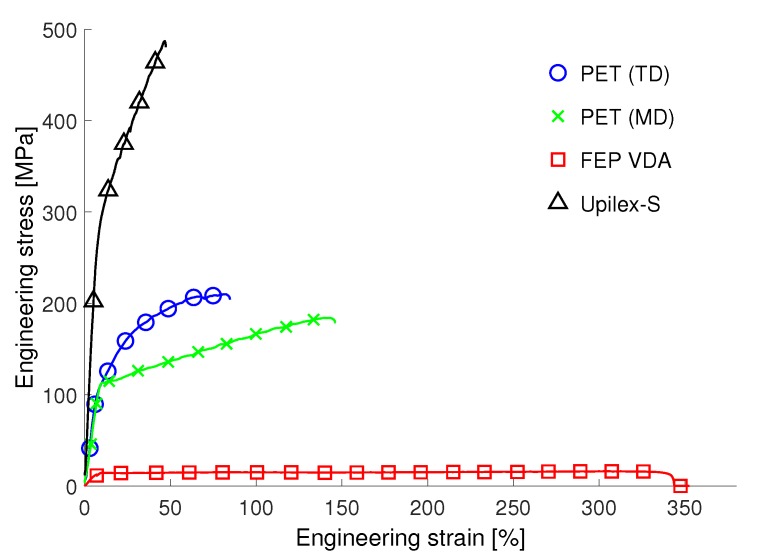
Results of the tensile experiments on non-irradiated FEP VDA, PET and Upilex-S VDA micro-samples. The materials show a large variation in their tensile behaviour. Additionally, the PET foils exhibit a distinct orientation dependency.

The tensile properties of the three investigated non-irradiated materials show large differences in both, the maximum strength and the maximum elongation (see Figure 6). While Upilex-S VDA shows a very high ultimate tensile strength (UTS), the FEP VDA foils exhibit low UTS and a considerably maximum tensile strain. The values for the PET samples lie within this range and exhibit additionally a distinctive orientation dependency. The directions of the orientation are commonly referred to as machine direction (MD) and transverse direction (TD) related to the fabrication procedure of the foil [29]. This orientation dependence has not been found for the Upilex-S VDA and FEP VDA foils. The resulting curves of Upilex-S VDA and PET are comparable to results of macroscopic experiments (according to ASTM D 882) [11,23]. The stress-strain curves measured for the FEP VDA samples exhibit a yield stress and a ultimate tensile strain in good agreement with macroscopic results [2]. However, the UTS from the micro-tests is significantly lower than the macroscopic value. The FEP VDA micro-samples do not show an increase of strength at elongation values of about 200% as reported for non-irradiated macroscopic specimens [2]. This suggests an alteration of the mechanisms responsible for the typical strain hardening of this material, due to the fs-laser.

### 3.3. Influence of Electron Beam Irradiation

#### 3.3.1. Upilex-S VDA

**Figure 7:**
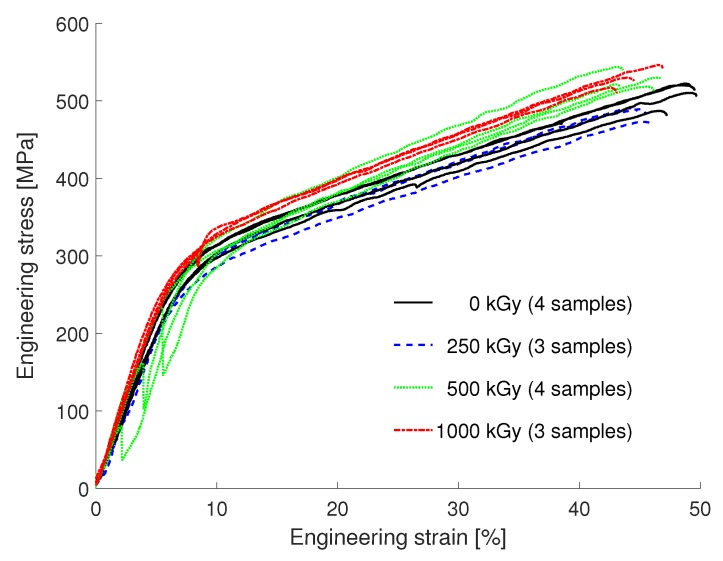
Results of the tensile experiments of the Upilex-S VDA foils exposed to irradiation doses of 0 kGy, 250 kGy, 500 kGy and 1000 kGy. The number of tested samples for each irradiation dose is indicated. There is no significant influence of the irradiation dose up to 1000 kGy. The deviation from a straight line in the linear elastic part shown by three curves, stems from a small rotation of the sample head due to a non-proper alignment with the gripper at the beginning of the tensile experiment.

The resulting tensile curves of Upilex-S VDA samples are shown in Figure 7. Foils irradiated with a dose of 0 (four samples), 250 (3), 500 (4) and 1000 (3) kGy were tested. No necking is observed during elongation. The experiments do not show a significant influence of the electron irradiation on the tensile properties. This high resistivity of Upilex against degradation by means of electron irradiation has already been shown [4,8,30]. No significant differences were found for the mechanical properties regarding the orientation of the samples (MD, TD), which has also been found in [4]. The results yield a very good agreement with the mechanical properties found in [23] (UTS of 520 MPa and maximum strain of 42%).

#### 3.3.2. PET

**Figure 8:**
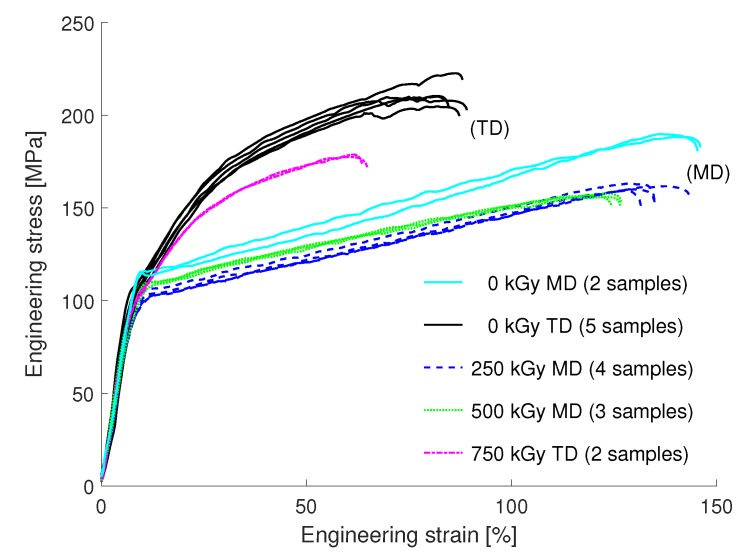
Results of the tensile experiments of the PET foils exposed to different irradiation doses. The number of tested samples for each irradiation dose is indicated. A reduction of the UTS was found for both, the machine and the transverse direction for all irradiation doses. For a dose of 750 kGy the maximum strain exhibited a significant reduction for the TD direction, respectively for the MD direction and 500 kGy. Only a slight reduction was found for the MD direction when irradiated with 250 kGy.

The stress-strain curves of the tensile experiments on the PET foils are shown in Figure 8. The PET foils exhibited a distinct dependence on the foil orientation (TD and MD). Both directions had a yield stress of about 105MPa, which agrees well with the values found in [31]. The UTS of the MD orientation is lower than that of the TD. However, a stronger increase of stress after the yield point is observed in the presented micro-tests compared to literature. This can be most likely linked to the higher strain rate used in the present work compared to the study of Poluektov et al. [31].

Regarding the electron irradiation samples with a dose of 0 kGy (5 samples TD, 2 MD), 250 kGy (4 MD), 500 kGy (3 MD) and 750 kGy (2 TD) were tested. Electron irradiation lead to a slight reduction of the yield strength. A significant decline in the maximal tensile strain and stress was found for both orientations. This is in contrast to the results in [32], which suggest for irradiation doses of 250 kGy and 500 kGy relatively constant values compared to non-irradiated PET samples.

#### 3.3.3. FEP VDA

**Figure 9:**
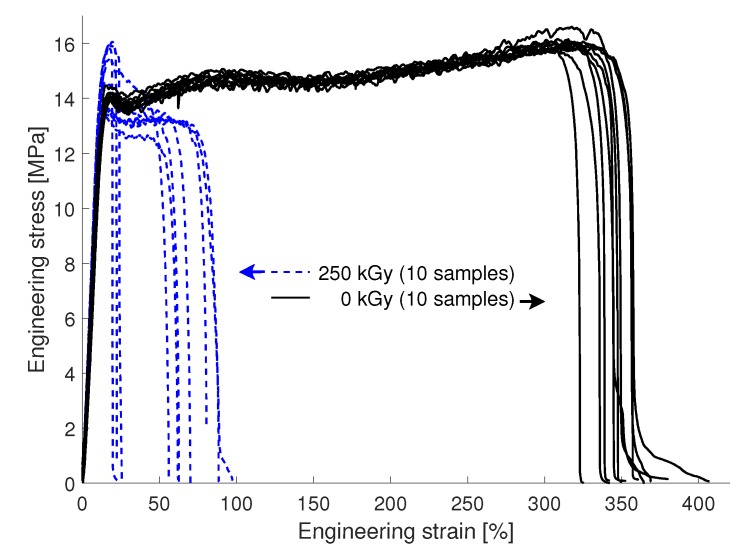
Results of the tensile experiments of the FEP VDA foils exposed to electron irradiation doses of 0kGy and 250kGy. For each irradiation dose 10 samples have been tested. The irradiated samples show a reduction of the ultimate tensile strain to about 7–25% of the value of non-irradiated samples. Furthermore, the irradiated samples exhibit an increased scatter of the yield strength.

In contrast to the Upilex-S VDA foils the FEP VDA foils are strongly affected by the electron irradiation. Figure 9 shows the results of tensile tests on 10 non-irradiated and 10 irradiated samples. Contrary to PET and Upilex-S VDA, the FEP VDA samples exhibit a load decrease after yielding, related to necking. In the case of the pristine samples the ultimate tensile strain yielded a mean value of 346% ± 11%, which agrees well with the value of 356% ± 8% given in [2]. The irradiation dose of 250 kGy leads to a reduction of this mean value to 57% ± 27%. No dependence of the resulting curves on the MD and TD direction was observed for the FEP VDA specimens.

Further the yield strength of 14.2±0.2MPa reported in [2] is in excellent agreement with the results in Figure 9. For the irradiated material an increase in the variation of the yield strength is observed. This increase is observed between the different sets of samples (four samples mounted on one clamp, see Section 2.2.1), while the scatter between the samples on a single mount stays in the same range as the value of the non irradiated specimens. As the positions of each sample set are located in a range of a few centimeters, this increased scatter in the yield strength possibly results from a local variation in the degradation due to a lateral variation of the electron irradiation dose.

### 3.4. Influence of fs-Laser Processing Atmosphere

The fs-laser processing atmosphere can play a critical role. Using atmospheric conditions the high energy density in the focal spot can lead to an ionization of the surrounding air [33]. In a study on PTFE [34] it has been shown that exceeding the threshold for ionization leads to a reduction of the ablation efficiency. Furthermore, the quality of processed surfaces decreases and an increase in debris deposition was found. To investigate the influence of atmospheric conditions during processing on the mechanical properties, tensile samples were cut with increasing fluence values of 1.18, 1.31, 1.96 and 2.62 J/cm${}^{2}$ under both, vacuum and ambient atmosphere. All other parameters were kept the same as listed in Table 2.

**Figure 10:**
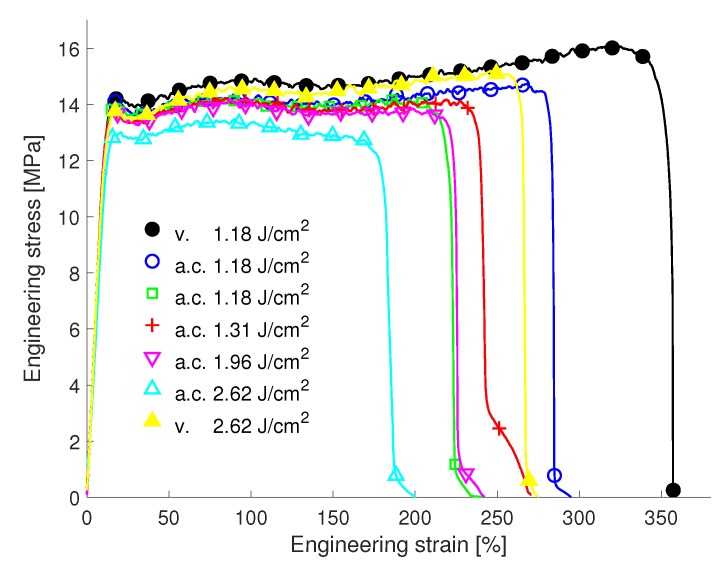
Influence of the ambient processing conditions on the tensile experiments of FEP VDA. For each condition, except the standard processing condition (vacuum (v.) and 1.18 J/cm${}^{2}$), one sample was tested. Processing under atmospheric conditions (a.c.) shows a somewhat smaller UTS and a significant reduction of the ultimate tensile strain compared to vacuum conditions. This effect increases with increasing fluence values.

For the Upilex-S VDA and PET micro-samples, processed under ambient pressure conditions, no change in tensile properties for increasing fluence values was found (see Appendix A). The FEP VDA samples, however, exhibited a significant reduction of the ultimate strain as shown in Figure 10. The curve for the sample processed with 1.18 J/cm${}^{2}$ under vacuum showed a maximum strain of about 350%. Increasing the fluence value to 2.62 J/cm${}^{2}$ reduces the ultimate strain also for vacuum conditions. This can be linked to a reduced quality of the cut and the occurrence of pores, which act as initial sites of failure. Fabricating the specimens under ambient air pressure, however, yielded a more pronounced reduction of the ultimate tensile strain for all fluence values. Additionally, the yield strength as well as the UTS is lowered for the samples processed under atmospheric conditions. Such an influence of the exposure environment on the extent of degradation of FEP has also been found for other types of irradiation and is linked to a higher yield of scission when irradiated in air [21].

An effect of using atmospheric processing conditions, as well as increasing fluence values was not found for irradiated FEP VDA samples. In this case, the influence of the electron irradiation dominates the decrease of the UTS and the maximum strain.

## 4. Outlook—Local Thinning of the Polymer Foil

Due to the very high energy electron beam used in this study and the small sample thickness, a constant electron irradiation damage across the thickness of the foils can be assumed. This will not be the case for foils used in space missions, hence, these samples may exhibit a gradient in irradiation damage across the foil thickness. A technique to evaluate the extent of this gradient as well as its influence on the mechanical properties has not been established yet. Due to the high material removal rate combined with the high precision, the fs-laser system allows to locally thin such foil samples. Taking into account the accuracy of the sample positioning, the thickness of the sample can be gradually reduced with a step-size of about 20μm.

**Figure 11:**
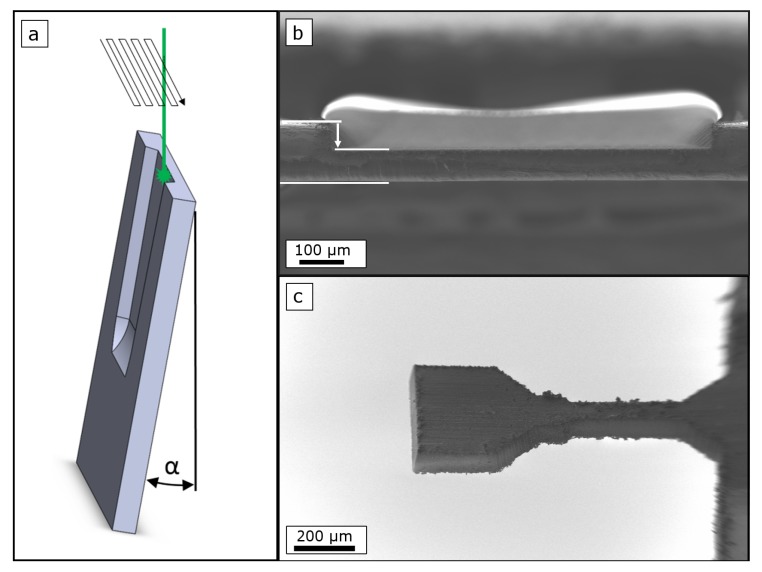
Fs-laser processing allows a local reduction of the foil thickness. (**a**) The thinning needs to be performed under the resulting, parameter dependent taper angle α. (**b**) FEP VDA foil thin section with a thickness of ca. 80 μm. (**c**) Tensile specimen cut into a thinned section.

The necessary preparation geometry is sketched in Figure 11a. The foil surface is oriented under an angle, which corresponds to the resulting taper angle, relative to the incidence direction of the laser. This arrangement allows to fabricate a trench with a sidewall parallel to the opposite foil surface. For this the laser beam is scanned in a meander-like path towards the intended depth in multiple repetitions. As the achievable resolution is about 20μm, linked to the positioning accuracy and the spot size, this approach is of interest for a foil thickness starting around 100μm. An example of a locally thinned FEP VDA foil with a thickness of 80μm is shown in Figure 11b, viewed from the laser incidence direction. To cut this trench the foil was positioned under an angle of about $\alpha =9^{\circ }$. This corresponds to the taper angle resulting when using the parameters for FEP VDA given in Table 2. An example of a tensile specimen cut into the locally thinned area is shown in Figure 11c. The tensile sample processing followed the fabrication route outlined in Section 2.2.1.

## 5. Conclusions

Femtosecond laser processing of polymer micro-samples was demonstrated for Upilex-S VDA, FEP VDA and PET foils. The structural quality of the samples is strongly influenced by the femtosecond laser pulse repetition rate due to heat accumulation effects. A repetition rate of 1 kHz yielded a minimal influenced zone, while ensuring a sufficient material removal rate.

The evaluation of tensile properties by means of the femtosecond laser processed micro-samples shows a good agreement with macroscopic data found in literature. No degradation of the samples due to laser processing was found for the PET and Upilex-S VDA specimens. However, the FEP VDA samples exhibited a sensitivity of the tensile properties on the ambient atmospheric conditions as well as the fluence of the fs-laser. Electron irradiation up to 1000 kGy did not influence the tensile properties of Upilex-S VDA foils. PET foils revealed a distinct orientation dependence and showed a minor degradation due electron beam irradiation, observable for all investigated doses from 250 to 750 kGy. FEP VDA foils exhibited a pronounced degradation already for an irradiation dose of 250 kGy. Besides a lower ultimate tensile strength, the ultimate tensile strain was reduced by a factor of about 6.

The study showed that femtosecond laser processing allows a fast and reproducible fabrication of polymer micro-samples with sizes of up to multiple hundreds of micrometers, therefore, enabling a high resolution for the investigation of local mechanical properties of polymer foils.

## Figures and Tables

**Table 1:** The investigated electron irradiation doses for each of the 3 different polymer foil materials.

Irradiation Dose [kGy]	0	250	500	750	1000
Upilex-S VDA	∘	∘	∘		∘
PET	∘	∘	∘	∘	
FEP VDA	∘	∘			

**Table 2:** Laser parameters used for the processing of the tested tensile samples in different foil materials. The respective total processing time and the resulting taper angle are given.

	Upilex-S	FEP	PET
	VDA	VDA	
Fluence [J/cm${}^{2}$]	0.65	1.18	1.18
Pulse rep. rate [kHz]	1	1	1
Scan speed [mm/s]	2	2	2
Scan repetitions [−]	15	40	40
Processing time [s]	≈380	≈1000	≈1000
Taper angle [${}^{\circ }$]	15.1±0.4	9.5±0.8	8.0±0.5

**Figure A1:**
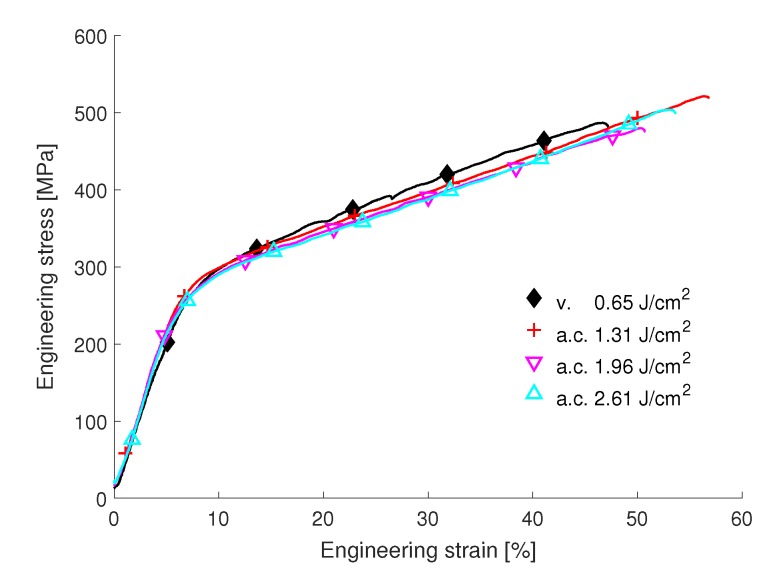
Influence of the ambient processing conditions on the tensile experiments of Upilex VDA foils. For atmospheric conditions (a.c.) and each fluence value one sample was tested. Comparing samples processed with increasing fluence under a.c. with samples processed under vacuum conditions (v.) does not reveal any influence. The scatter of the data lies within the scatter observed in Figure 7.

**Figure A2:**
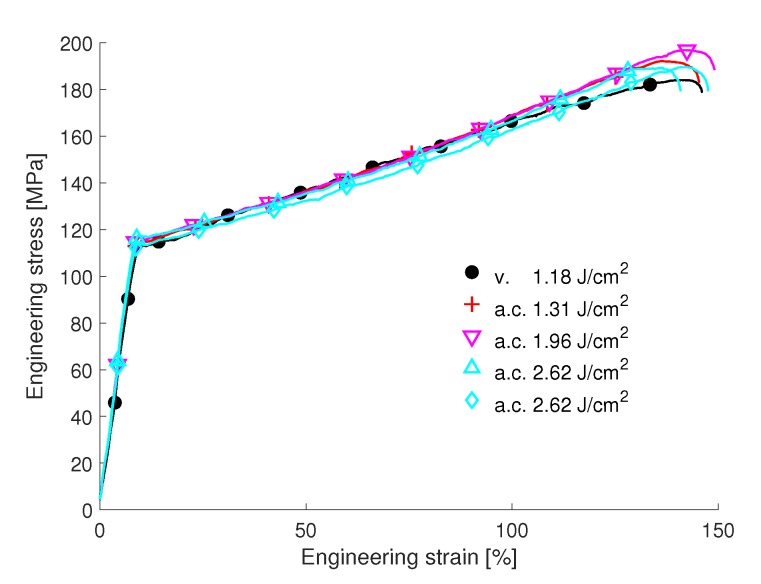
Influence of the ambient processing conditions on the tensile experiments of PET foils in MD. For atmospheric conditions (a.c.) and fluence value of 1.31 and 1.96 J/cm${}^{2}$ one sample was tested. For the fluence of 2.62 J/cm${}^{2}$ two curves are given. Comparing samples processed with increasing fluence under a.c. with samples processed under vacuum conditions (v.) does not reveal any influence. The scatter of the data lies within the scatter observed in Figure 8.

## Appendix A

The influence of the atmospheric conditions during laser processing on the tensile properties was found to be significant for the FEP VDA foils, exhibiting a distinct decrease in the ultimate tensile strain (see Section 3.4). For non-irradiated Upilex VDA foils (Figure A1) and non-irradiated PET foils (Figure A2) no such effect was found. For both materials, even for the highest fluence values, the ultimate tensile stress and the ultimate tensile strain are within the scatter observed for samples processed under vacuum conditions.

## References

[1] Townsend J.A., Hansen P.A., Dever J.A., de Groh K.K., Banks B.A., Wang L., He C. (1999). Hubble Space Telescope Metallized Teflon^®^ FEP thermal Control Materials: On-Orbit Degradation and Post-Retrieval Analysis. High Perform. Polym..

[2] Townsend J.A., Powers C.E., Viens M.J., Ayres-Treusdell M.T., Munoz B.F. Degradation of Teflon^®^ FEP Following Charged Particle Radiation and Rapid Thermal Cycling. Proceedings of the 20th Space Simulation Conference.

[3] Semprimoschnig C.O.A., Heltzel S., Polsak A., Van Eesbeek M. (2004). Space Environmental Testing of Thermal Control Foils at Extreme Temperatures. High Perform. Polym..

[4] Sasuga T., Hayakawa N., Yoshida K., Hagiwara M. (1985). Degradation in tensile properties of aromatic polymers by electron beam irradiation. Polymer.

[5] Dever J.A., de Groh K.K., Banks B.A., Townsend J.A. (1999). Effects of Radiation and Thermal Cycling on Teflon^®^ FEP. High Perform. Polym..

[6] Moser M., Ranzenberger C., Duzellier S. (2016). Space Environmental Testing of Novel Candidate Materials for Multilayer Insulation. J. Spacecr. Rocket..

[7] Moser M., Semprimoschnig C.O.A., Van Eesbeek M., Pippan R. Comparison of results from post-flight investigations on FEP retrieved from the Hubble Space Telescope solar arrays and LDEF. Proceedings of the European Conference on Spacecraft Structures.

[8] Shimamura H., Yamagata I. (2009). Degradation of Mechanical Properties of Polyimide Film Exposed to Space Environment. J. Spacecr. Rocket..

[9] Chen J., Ding N., Li Z., Wang W. (2016). Organic polymer materials in the space environment. Prog. Aerosp. Sci..

[10] Dever J.A., Miller S.K., Sechkar E.A., Wittberg T.N. (2008). Space Environment Exposure of Polymer Films on the Materials International Space Station Experiment: Results from MISSE 1 and MISSE 2. High Perform. Polym..

[11] ASTM (2018). ASTM D882-18, Standard Test Method for Tensile Properties of Thin Plastic Sheeting.

[12] Nolte S., Momma C., Jacobs H., Tünnermann A., Chichkov B.N., Wellegehausen B., Welling H. (1997). Ablation of metals by ultrashort laser pulses. J. Opt. Soc. Am. B.

[13] Shirk M.D., Molian P.A. (1998). A review of ultrashort pulsed laser ablation of materials. J. Laser Appl..

[14] Krüger J., Kautek W., Lippert T.K. (2004). Ultrashort Pulse Laser Interaction with Dielectrics and Polymers. Polymers and Light.

[15] Mohd Abbas N., Solomon D.G., Fuad Bahari M. (2007). A review on current research trends in electrical discharge machining (EDM). Int. J. Mach. Tools Manuf..

[16] Chichkov B.N., Momma C., Nolte S., Alvensleben F.V., Tünnermann A. (1996). Femtosecond, picosecond and nanosecond laser ablation of solids. Appl. Phys. A.

[17] ASM International (2013). ISTFA 2013: Proceedings from the 39th International Symposium for Testing and Failure Analysis.

[18] Slaughter S.K., Ligda J.P., Sano T., Schuster B.E. (2015). High Throughput Femtosecond-Laser Machining of Micro-Tension Specimens. Proceedings of the TMS 2015 144th Annual Meeting & Exhibition.

[19] Jakob S., Pfeifenberger M.J., Hohenwarter A., Pippan R. (2017). Femtosecond laser machining for characterization of local mechanical properties of biomaterials: A case study on wood. Sci. Technol. Adv. Mater..

[20] Magagnosc D.J., Ligda J.P., Sano T., Schuster B.E. (2018). Femtosecond Laser Machining of Micro-tensile Specimens for High Throughput Mechanical Testing. Micro and Nanomechanics.

[21] de Groh K.K., Banks B.A., Miller S.K.R., Dever J.A., Kutz M. (2018). Chapter 28—Degradation of Spacecraft Materials. Handbook of Environmental Degradation of Materials.

[22] Pfeifenberger M.J., Mangang M., Wurster S., Reiser J., Hohenwarter A., Pfleging W., Kiener D., Pippan R. (2017). The use of femtosecond laser ablation as a novel tool for rapid micro-mechanical sample preparation. Mater. Des..

[23] Yang S.Y. (2018). Advanced Polyimide Materials: Synthesis, Characterization, and Applications.

[24] de Groh K.K., Waters D., Mohammed J., Perry B., Banks B. (2013). Analyses of Hubble Space Telescope Aluminized-Teflon Insulation Retrieved After 19 Years of Space Exposure. Protection of Materials and Structures From the Space Environment, Astrophysics and Space Science Proceedings.

[25] Kumagai H., Midorikawa K., Toyoda K., Nakamura S., Okamoto T., Obara M. (1994). Ablation of polymer films by a femtosecond high peak power Ti:sapphire laser at 798 nm. Appl. Phys. Lett..

[26] Yang B., Motz C., Grosinger W., Kammrath W., Dehm G. (2008). Tensile behaviour of micro-sized copper wires studied using a novel fibre tensile module. Int. J. Mater. Res..

[27] Nolte S., Schrempel F., Dausinger F. (2015). Ultrashort Pulse Laser Technology: Laser Sources and Applications.

[28] Eaton S.M., Zhang H., Herman P.R., Yoshino F., Shah L., Bovatsek J., Arai A.Y. (2005). Heat accumulation effects in femtosecond laser-written waveguides with variable repetition rate. Opt. Express.

[29] Michler G.H., Baltá Calleja F.J. (2012). Nano- and Micromechanics of Polymers: Structure Modification and Improvement of Properties.

[30] Semprimoschnig C.O.A., Gray P., Nehls M.K., Edwards D.L. Accelerated space environmental testing and analysis of ultra-thin polymer films for Gossamer space structures like solar sails. Proceedings of the 10th International Symposium on Materials in a Space Environment.

[31] Poluektov M., Dommelen J.A.W., Govaert L.E., Geers M.G.D. (2014). Characterisation and modelling of anisotropic thermo-mechanical behaviour of oriented polyethylene terephthalate. Model. Simul. Mater. Sci. Eng..

[32] Źenkiewicz M. (2004). Effects of electron-beam irradiation on some mechanical properties of polymer films. Radiat. Phys. Chem..

[33] Wang Z.B., Hong M.H., Lu Y.F., Wu D.J., Lan B., Chong T.C. (2003). Femtosecond laser ablation of polytetrafluoroethylene (Teflon) in ambient air. J. Appl. Phys..

[34] Wang Y., Wang X., Zhang N., Zhai H., Zhu X. (2005). Study of ambient air ionization with femtosecond laser pulses. Proc. SPIE.

